# Preparing for an orthopedic consultation using an eHealth tool: a randomized controlled trial in patients with hip and knee osteoarthritis

**DOI:** 10.1186/s12911-020-01130-0

**Published:** 2020-05-15

**Authors:** Aniek A. O. M. Claassen, Henk J. Schers, Vincent J. J. F. Busch, Petra J. C. Heesterbeek, Frank H. J. van den Hoogen, Thea P. M. Vliet Vlieland, Cornelia H. M. van den Ende

**Affiliations:** 1 Department of Rheumatology, Sint Maartenskliniek, PO Box 9011, Nijmegen, GM 6500 The Netherlands; 2 Department of Primary and Community Care, Radboud University Medical Center, Nijmegen, The Netherlands; 3 Department of Orthopaedic Surgery, Sint Maartenskliniek, Nijmegen, The Netherlands; 4 Sint Maartenskliniek Research, Sint Maartenskliniek, Nijmegen, The Netherlands; 5 Department of Rheumatology, Radboud University Medical Center, Nijmegen, The Netherlands; 6 Department of Orthopaedics, Rehabilitation and Physical Therapy, Leiden University Medical Center, Leiden, The Netherlands

**Keywords:** Hip and knee osteoarthritis, eHealth, Consultation, Preparation, Education

## Abstract

**Background:**

To evaluate the effect of a stand-alone mobile and web-based educational intervention (eHealth tool) compared to usual preparation of a first orthopedic consultation of patients with hip or knee osteoarthritis (OA) on patients’ satisfaction.

**Methods:**

A two-armed randomized controlled trial involving 286 patients with (suspicion of) hip or knee OA, randomly allocated to either receiving an educational eHealth tool to prepare their upcoming consultation (*n* = 144) or usual care (*n* = 142). Satisfaction with the consultation on three subscales (range 1–4) of the Consumer Quality Index (CQI - primary outcome) and knowledge (assessed using 22 statements on OA, range 0–22), treatment beliefs (assessed by the Treatment beliefs in OsteoArthritis questionnaire, range 1–5), assessment of patient’s involvement in consultation by the surgeon (assessed on a 5-point Likert scale) and patient satisfaction with the outcome of the consultation (numeric rating scale), were assessed.

**Results:**

No differences between groups were observed on the 3 subscales of the CQI (group difference (95% CI): communication 0.009 (− 0.10, 0.12), conduct − 0.02 (− 0.12, 0.07) and information provision 0.02 (− 0.18, 0.21)). Between group differences (95% CI) were in favor of the intervention group for knowledge (1.4 (0.6, 2.2)), negative beliefs regarding physical activities (− 0.19 (− 0.37, − 0.002) and pain medication (− 0.30 (− 0.49, − 0.01)). We found no differences on other secondary outcomes.

**Conclusions:**

An educational eHealth tool to prepare a first orthopedic consultation for hip or knee OA does not result in higher patient satisfaction with the consultation, but it does influence cognitions about osteoarthritis.

**Trial registration:**

Dutch Trial Register (trial number NTR6262). Registered 30 January 2017.

## Background

Non-surgical treatments like lifestyle education, exercise therapy, weight loss and pain medication are recommended as a primary approach to manage hip or knee osteoarthritis (OA) in an early stage and can be organized in primary care [1, 2]. Once these conservative treatment options have been tried adequately and have failed, or in case of diagnostic uncertainty, referral to an orthopedic surgeon should be considered for further evaluation and consideration of surgical interventions, e.g. a total joint replacement (TJR) [2]. Among medical, economic and healthcare professionals’ factors, patient-related factors may influence the choice for a TJR [3]. To actively participate in the consideration of different treatment options, patients need to be informed of benefits and possible disadvantages of available treatment options [4].

Currently, half to two-third of patients referred to an orthopedic surgeon are considered not (yet) eligible for a TJR [5, 6]. This is in contrast with the observation that patients with hip or knee OA who are referred for a first orthopedic consultation often expect action to be taken [7], in particular the planning of a TJR. It is conceivable that expectations for some patients are not met, resulting in patients being dissatisfied [8].

An appropriate preparation of the consultation is likely to streamline the patients’ expectations and increase their satisfaction, irrespective of whether the outcome is consideration of surgery or not. This hypothesis is supported by the literature where, in general, patients who are more knowledgeable, skilled and proactive prior to a consultation are more satisfied with received care because it is more likely that needs are met [9]. Interventions aimed at supporting patients to prepare for a consultation were found to improve self-efficacy in older patients [10]. Moreover, educational tools have high satisfaction rates and positive effects on patient knowledge, decision making, self-efficacy and number of questions asked during consultation [11, 12].

An educational eHealth application may be suitable to prepare patients for their consultation because of the easy accessibility and the possibility to provide information that suits individual preferences and needs. Moreover, eHealth interventions have shown to enhance and supplement the communication between patients and healthcare providers and seem effective at providing information, enhancing information exchange, and promoting self-management in older adults [13, 14]. Recently it was concluded that the use of an educational website for patients with hip and knee OA improve important aspects of quality of care (i.e. self-management, lifestyle and physical activity) [15]. However, these results were based on an observational study and to our knowledge good quality randomized controlled trials evaluating educational eHealth tools with interactive parts are not available.

The aim of the present study was to evaluate the effect of a stand-alone mobile and web-based educational intervention (educational eHealth tool) compared to usual preparation of a first orthopedic consultation of patients with hip or knee OA on satisfaction with the consultation. Secondary outcomes were knowledge, treatment beliefs and measures on the consultation from the patient and surgeon’s perspective.

## Methods

### Design and setting

This study was reported according to the CONSORT guidelines [16]. A two-armed unblinded randomized controlled trial was conducted. The study was performed at the outpatient departments of Orthopaedic surgery of the Sint Maartenskliniek Nijmegen and Boxmeer, the Netherlands from March 2017 to May 2018. The need for ethics approval was waived. The local Medical Research Ethics Committee, region Arnhem-Nijmegen (study no. 2016–3096) provided a waiver, as this type of study does not require approval from an ethics committee in the Netherlands according to the Central Committee on Research involving Human Subjects. The study was registered in the Dutch Trial Register (trial number NTR6262). All participants gave informed consent prior to the baseline data collection*.*

### Participants

Patient with hip or knee OA with a scheduled first-time visit for a new diagnosis at the outpatient department of Orthopaedic surgery of the Sint Maartenskliniek were checked for eligibility. Patients were invited for participation when: 1) 18 years or older; 2) diagnosis or suspicion of OA in the knee or hip in the referral letter; and 3) no previous visit to the outpatient department of Orthopaedic surgery of the Sint Maartenskliniek for a complaint of the index joint. Exclusion criteria were: 1) unable to read and understand the Dutch language; 2) not possessing a smartphone, computer or tablet; or 3) no e-mail address.

### Interventions

The **intervention** and **control** group received the usual hospital procedure. Participants received a confirmation letter of their scheduled consultation along with a flyer named “Going prepared to the outpatient department of Orthopaedic surgery”. This flyer provides brief information on how to prepare for the consultation in addition to practical information regarding the visit to the hospital.

The **intervention** group also received a login to access the educational eHealth tool no more than two weeks before their consultation. The educational eHealth tool was developed following an iterative method of persuasive design in collaboration with OA patients [17]. Pilot-testing of the developed tool was done among patients and healthcare professionals. The tool could be consulted using a smartphone, a tablet or computer. The tool contained the following functionalities: (1) information on OA and treatment modalities, based on a stepped-care strategy for OA [18]; (2) preparation for the upcoming consultation consisting of predefined questions to answer, and space to record questions the patient would like to ask the orthopedic surgeon; (3) the option to monitor pain and fatigue during 1 week prior to the consultation; (4) list medication use with the option to set reminders for intake; and (5) the option to create a visual timeline with the scheduled consultation, assessments and preparation. Further specifications and the developmental process of the tool are described elsewhere [19].

### Procedures

**Fig. 1:**
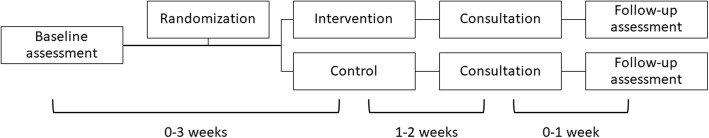
Study protocol and timeline for participants

Eligible patients received an information letter to participate. Patients were invited based on the referral letter of the general practitioner or referring specialist, which was screened by a research assistant on confirmed diagnosis or suspicion of knee or hip OA. Patients willing to participate were asked to contact the involved researcher. After registering for the study, participants received information about the study by e-mail, along with a hyperlink to an online consent form and questionnaire for baseline assessment (T0). Once the questionnaire was completed, participants were allocated to the intervention or control group (concealed allocation ratio 1:1, stratified by main OA-location hip or knee, using randomly varied block sizes (4 to 8)). Randomization was performed using an electronic data capture and management program; Castor EDC (www.castoredc.com). Participants were informed of the allocation through email, intervention group participants also received access to the educational eHealth tool. One day after the consultation all participants received the second questionnaire (T1) through email. Non-responding participants received a reminder after 1 week. The timeline for participants is illustrated in Fig. 1. Participants who did not attend the consultation were excluded. Diagnosis of all participants was checked in the patient information system post-consultation. Directly following the consultation the orthopedic surgeon rated the degree of involvement of the patient. All data were collected or processed in Castor EDC.

### Measurements and outcomes

Data on demographic (gender, age, BMI, level of education, work status) and clinical (OA index joint, number of painful joints, duration of symptoms, pain and function) characteristics were collected at baseline (T0), 2–5 weeks prior to the consultation. To asses pain and function the Western Ontario and McMaster Universities Osteoarthritis Index (WOMAC) was used [20], with standardized scores being presented (0–100, higher scores indicating more pain and worse function). Primary and secondary outcome measures were collected during the week after the consultation (T1).

#### Primary outcome measure

The primary outcome was satisfaction with the consultation measured with an adapted version of the Consumer Quality Index (CQI), the Dutch standard for measuring patient experience with health care providers and health plans [21]. Because the CQI is available for several curative services, but not specifically for OA or a visit to an orthopedic surgeon, we adapted three subscales of the CQI, 1) The subscale “physician-patient communication” from the ‘CQI-general practitioner care’ with the addition of 2 items from the ‘CQI-outpatient clinic’. 2) the subscale “conduct physician” from the ‘CQI-Rheumatoid Arthritis’ supplemented with two items from the ‘CQI-general practitioner care’ and 3) the subscale ‘“Information provision by the physician” from CQI-outpatient clinic’. The three subscales are independently validated for the three mentioned CQI-indices [22–24]. For each subscale an indicator score can be calculated ranging from 1 to 4 (higher score indication higher satisfaction with care).

#### Secondary outcome measures

To evaluate the consultation several self-administered questions were used. *Treatment strategy after the consultation* was asked by means of the question: “What did you and the doctor agreed on to do next?” (the doctor referred me to another healthcare professional, namely …. / I’m getting surgery / the doctor described pain medication / I don’t know / We did not agree on a next step / wait and see / other, namely…). Referrals to other healthcare professionals (e.g. physiotherapists or dieticians) as well as answers in the “other”-category that comprised recommendations on physical exercise or dieting were categorized as “conservative treatment strategy”. *Satisfaction* with the consultation and the policy after the consultation could be scored on a Numeric Rating Scale (NRS) (0–10).

Based on frequently asked questions on OA in a previous study [25], 22 statements were self-administered to assess *knowledge* of participants on OA (treatment) at baseline (T0) and follow-up (T1). Total score ranged from 0 to 22, with higher scores indicating more knowledge. To asses patients’ thoughts and expectations regarding treatment options (physical activities, pain medication and joint replacement surgery) the Treatment beliefs in OsteoArthritis questionnaire (TOA) was used [26]. Positive and negative *treatment beliefs* were measured at baseline (T0) and follow-up (T1). To facilitate interpretation mean subscale scores were divided by the number of items per scale, resulting in a standardized score ranging from 1 to 5 (1 = less positive, 5 = more positive [positive subscales] and 1 = less negative, 5 = more negative [negative subscales). The TOA shows good internal consistency and reliability [26].

Orthopedic surgeons were asked to score two statements about the consultation on a 5-point Likert scale (1 “completely disagree” – 5 “completely agree”): 1) “the patient showed to be well prepared for the consultation” and 2) The patient had an active role in the consultation”.

For the purpose of a usability study on the educational eHealth tool, participants in the intervention group were asked to fill in several additional questions in the follow-up measure [19].

### Statistical analysis

Based on previous research [27] and collected unpublished data on the CQI in the Sint Maartenskliniek an a-priori sample size estimate indicated that 286 participants (143 per group) would provide 80% power at 5% level of significance (two-sided) to detect a treatment difference of at least 0.15 points on the CQI subscales between the two groups assuming a SD of 0.45. Accounting for 25% loss of follow up we aimed to include 382 patients.

Data were analyzed using Stata 13.1. Primary analysis were done according to the intention-to-treat (ITT) principle. Secondary analyses included per-protocol analysis excluding protocol violators (i.e. patients who did not open the educational eHealth tool, based on log-file analysis). Additionally, we analyzed differences in satisfaction between patients of whom the outcome of the consultation was surgery and patients with a different outcome of the consultation.

Post-intervention differences between groups were analyzed using linear regression analyses, Chi-squared test and Mann-Whitney U test where appropriate. Data on knowledge and treatment beliefs were analyzed with linear regression analyses, using follow-up scores as dependent variable and group (intervention/control) and baseline value as covariate. All linear regression analyses were corrected for outcome of consultation (surgery or not). Differences between groups and 95% CI were reported.

## Results

**Fig. 2:**
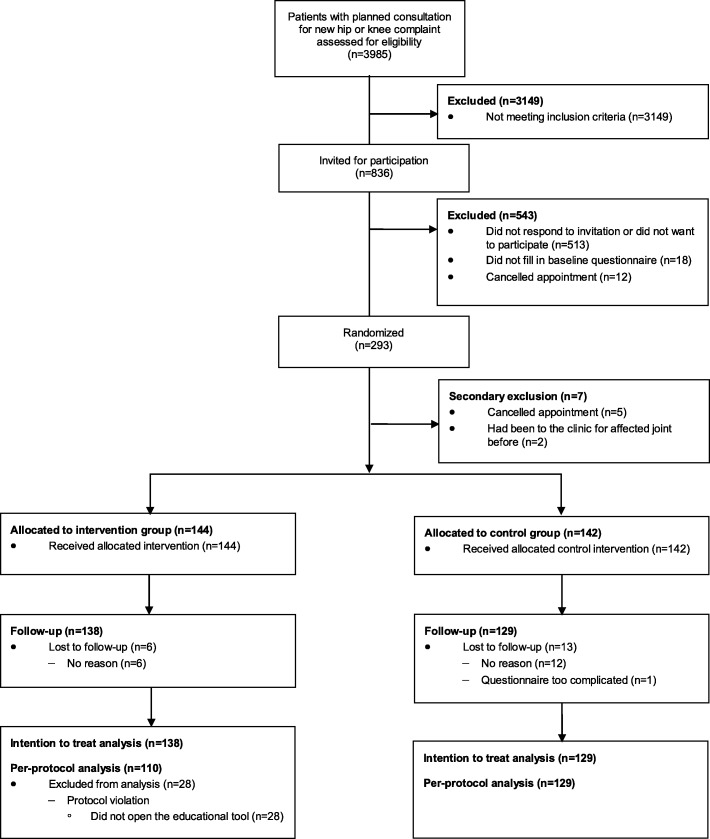
Flow diagram of patient inclusion in the trial

Between March 2017 and March 2018, 836 individuals were invited to participate. A total of 293 (35%) participants filled in the first questionnaire and were randomized. No differences were found between the invited patients who did not participate and the study population with regard to age (*P*-value = 0.08) and sex (*P*-value = 0.61). Due to time constraints inclusion was stopped after 293 out of the targeted 382 patients were enrolled. Data of 7 (2%) participants was excluded because they did not fulfil the inclusion criteria; 5 participants cancelled their appointment and 2 participants were wrongly included as it turned out they already had been to the clinic before for OA complaints in the same joint. Two hundred eighty-six participants were allocated to either the intervention (*n* = 144) or control (*n* = 142) group. Nineteen (7%) participants were lost to follow-up leaving data of 267 participants for the ITT analysis. Twenty-eight participants in the intervention group did not open the application and were considered protocol-violators and excluded in the per-protocol analysis (Fig. 2).

Baseline characteristics of the study population are shown in Table 1. The majority of patients was female (58%), around 80% of patients had a consultation with regard to complaints on the knee and more than half experienced their first symptoms in the previous 5 years.

**Table 1:** Baseline characteristics of participants allocated to the intervention and control group

	Intervention group (*n* = 144)	Control group (*n* = 142)
*Social-demographic characteristics*		
Female; n (%)	81 (57)	85 (60.7)
Age, years; mean (S.D.)	61.7 (10.4)	63.3 (10.1)
BMI, kg/m^2^; mean (S.D.)	27.9 (4.4)	29.0 (5.1)
< 12 years education; n (%)	57 (40)	56 (39.7)
Paid work; n (%)	58 (43)	47 (35.6)
*Clinical characteristics*		
Index joint knee; n (%)	115 (80)	112 (78.9)
Number of painful joints (0–10); median (IQR)	2 (1–3.5)	2 (1–4)
Duration of symptoms; n (%)		
< 1 year	14 (10)	19 (14)
1–5 years	69 (49)	64 (46)
5–10 years	20 (14)	22 (15)
> 10 years	39 (27)	35 (25)
Pain, WOMAC (0–100); mean (S.D.)	50.9 (19.8)	47.6 (19.1)
Function, WOMAC (0–100); mean (S.D.)	55.0 (21.1)	48.5 (20.5)

### Primary outcome

No relevant or significant differences between the intervention and control group were found on consultation satisfaction, as measured with all three subscales (communication, conduct and information provision) of the CQI (Table 2).

**Table 2:** Follow-up indicator scores and differences between groups on the subscales of the Consumer Quality Index

	Intervention group		Control group		Group difference
	n	Mean (S.D.)	n	Mean (S.D.)	Δ (95% CI)^a^
Communication, CQI^b^ (1–4)	129	3.69 (0.47)	118	3.66 (0.45)	0.009 (− 0.10, 0.12)
Conduct, CQI^b^ (1–4)	133	3.78 (0.40)	124	3.76 (0.41)	− 0.02 (− 0.12, 0.07)
Information provision, CQI^b^ (1–4)	80	3.59 (0.69)	90	3.58 (0.65)	0.02 (− 0.18, 0.21)

^a^ Adjusted for treatment strategy (surgery or not). ^b^ Adapted version. CQI: Consumer Quality Index

### Secondary outcomes

About one-fourth of the patients were put up for joint replacement surgery (Table 3). Twenty-two percent of patients in the intervention group and 29% in the control group were referred to or received a conservative treatment option (physiotherapy, dietary therapy, pain medication, brace, etc.). In another one-third it was decided to abide further symptom development.

**Table 3:** Differences on secondary outcomes of the consultation for the intervention group and control group

	Intervention group *n* = 138	Control group *n* = 129	*P*-value
*Patients’ outcomes*			
Able to ask what I wanted; n (%)	121 (88)	104 (81)	0.12^a^
Number of questions asked, median (IQR)	2 (1–3)	2 (1–3)	0.31^b^
Treatment strategy after consultation, n (%)			0.47^a^
Conservative	30 (22)	37 (29)	
Surgery	37 (27)	34 (26)	
Wait and see	46 (33)	34 (26)	
Other diagnosis	16 (12)	17 (13)	
Satisfaction with policy (0–10), mean (S.D.)	7.5 (2.7)	7.9 (2.2)	−0.4 (−1.1, 0.2)^c^
Satisfaction with consultation (0–10), mean (S.D.)	8.0 (2.3)	8.3 (2.0)	−0.2 (− 0.8, 0.4)^c^
*Surgeons’ outcomes*			
Preparedness of patient (1–5), median (IQR)	5 (4–5)	4 (4–5)	0.51^b^
Participation of patient (1–5), median (IQR)	5 (4–5)	5 (4–5)	0.82^b^

^a^ Chi-squared test. ^b^ Mann-Whitney U test. ^c^ Linear regression analysis, adjusted for treatment strategy (surgery or not), mean difference (95% CI)

Knowledge significantly improved in the intervention group compared to the control group (mean group difference (95% CI): 1.4 (0.6, 2.2)) (Table 4). Also, significant differences were found in negative beliefs regarding physical activities and pain medication between intervention and control group, with the intervention group having less negative beliefs (mean group difference (95% CI): − 0.19 (− 0.37, − 0.002) and − 0.30 (− 0.49, − 0.12) respectively). No other differences were found in any of the secondary outcome measures.

**Table 4:** Differences in knowledge and treatment beliefs for the intervention group and control group

	Intervention group		Control group		Group difference (95% CI)^a^
	Baseline mean (S.D.)	Follow-up mean (S.D.)	Baseline mean (S.D.)	Follow-up mean (S.D.)	
Knowledge (0–22)	11.2 (3.7)	12.9 (4.1)	11.2 (3.7)	11.6 (4.3)	1.4 (0.6, 2.2)*
Treatment beliefs, TOA (1–5)					
Positive – PA	3.63 (0.84)	3.88 (0.79)	3.46 (0.85)	3.77 (0.83)	0.004 (−0.16, 0.17)
Negative - PA	2.78 (1.00)	2.55 (0.92)	2.94 (0.98)	2.78 (0.91)	−0.19 (− 0.37, − 0.002)*
Positive – PM	3.35 (0.99)	3.63 (1.03)	3.24 (1.03)	3.58 (0.92)	−0.01 (− 0.16, 0.18)
Negative – PM	3.59 (0.81)	3.22 (0.82)	3.71 (0.74)	3.59 (0.66)	−0.30 (− 0.49, − 0.12)*
Positive – TJR	3.98 (0.70)	3.94 (0.68)	4.06 (0.65)	4.10 (0.63)	−0.10 (− 0.21, 0.01)
Negative – TJR	3.75 (0.79)	3.90 (0.73)	3.66 (0.84)	3.94 (0.73)	−0.08 (− 0.23, 0.06)

^a^ adjusted for baseline score of outcome (i.e. knowledge, TOA) and treatment strategy (surgery or not). *Significant for *P* < 0.05. TOA: Treatment Beliefs in Osteoarthritis questionnaire; PA: physical activities; PM: pain medication; TJR: joint replacement surgery

### Secondary analysis

The per-protocol analysis was performed excluding 28 patients from the intervention group, all whom did not open the application. Experience with the consultation in the intervention group was found not to be significantly different than in the control group on all three CQI subscales. Only small differences were found on secondary outcomes compared to the ITT-analysis. The decrease of negative beliefs regarding physical activities in favor of the intervention group was not found statistically significant any longer (mean group difference (95% CI: − 0.17 (− 0.36, 0.03)) and positive beliefs about total joint replacement surgery decreased in those who had used the intervention, compared to the control group (mean group difference (95% CI: − 0.12 (− 0.24, 0.001)).

Secondary analysis on satisfaction outcomes between patients scheduled for surgery versus those with a different treatment strategy revealed that patients scheduled for surgery were more satisfied as measured with the CQI subscales ‘Conduct’ and ‘Information provision’ (mean group difference (95% CI): 0.18 (− 0.29, − 0.07) and − 0.32 (− 0.53, − 0.11), respectively). Also, satisfaction with the treatment strategy and the consultation as measured on NRS (0–10) was significantly higher in patients scheduled for surgery (mean group difference (95% CI): − 1.1 (− 1.8, − 0.3) and − 1.1 (− 1.8, − 0.5) respectively).

## Discussion

The results of this study show that preparing a first orthopedics consultation for hip or knee OA using an educational eHealth tool does not result in higher satisfaction of the consultation. Participants using the eHealth tool did have more knowledge and less negative beliefs about physical activities and pain medication as compared to usual care. No other significant differences between intervention and control group were found regarding treatment strategy (surgery versus other strategies), or treatment beliefs about TJR. Neither were there differences between groups regarding the evaluation by the orthopedic surgeon on preparation and active participation of the consultation.

We hypothesized that patients are more satisfied with their consultation regardless of the chosen treatment when they are well prepared using an educational eHealth tool. The results do not confirm our hypothesis, but are in line with a recently published RCT demonstrating no improvement in the appreciation of the first orthopedic consultation, after the use of an educational smartphone app [28]. However, secondary analysis showed that patients with a TJR planned as result of the consultation were significantly more satisfied than patients who had a different outcome, irrespective of the use of the tool. This confirms findings from previous qualitative studies that patients want action to be taken [6] and prefer TJR as treatment [29]. Previous research shows that expectations of TJR are often high and not always realistic [30, 31], but are a key determinant of treatment satisfaction after TJR [32–34]. Also, patients’ beliefs about conservative and surgical treatments options are an important aspect in the choice for a treatment [3, 35]. Our results are in line with recent findings showing that educational tools used either during or in preparation for the consultation improve knowledge and risk perception [28, 36]. However, effects of improvement in knowledge and risk perception on fulfilment of expectations and ultimately satisfaction still has to be investigated [37].

The lack of positive effects of the tool on satisfaction may have several explanations. First, it may indicate that our intervention was not comprehensive enough to sufficiently change patients’ expectations towards other treatment options in order to improve their satisfaction. Although fulfilment of expectations is an important aspect of satisfaction [8, 33], healthcare professional related aspects like trust, empathy, communication and relatedness, contact time and waiting time are important for satisfaction with the consultation as well [8]. These aspects were not specifically targeted in our intervention. Second, in hindsight our choice for using the CQI as outcome measure may be questioned for several reasons. Because of missing values we could not calculate indicator scores for all participants. Moreover, it should be noted that satisfaction was high in all patients. Scores found in our study were even higher than previously reported CQI scores in orthopedics setting (3.3 (hospital stay) and 3.5 (information at discharge)) [27]. Moreover, > 15% of participants scored the highest possible CQI scores indicating a ceiling effect on our primary outcome [38]. The primary focus of the eHealth tool was to target patient expectations. Fulfilment of expectations was not reflected in the CQI and as such not measured. A validated sensitive questionnaire to assess satisfaction incorporating fulfilment of patient expectations about the consultations and its outcomes is needed, but currently unavailable.

Based on the positive effects on knowledge and beliefs found in this study, further implementation of the educational eHealth tool may be valuable for clinical practice. More guidance on the use of our educational eHealth tool and instruction for the orthopedic surgeon to discuss preparing the consultation may result in better outcomes [39] and should be a focus of further implementation. However, an advantage of eHealth interventions is that they can be used irrespective of time and place, without involvement of a healthcare professional. If the ultimate aim is to routinely use the educational eHealth tool, costs and benefits with regard to what is effective and what is practical should be weighted and further studied. Additionally, our intervention is already suited for preparation for consultation with other healthcare professionals (e.g. physiotherapist or general practitioner). It may therefore also be implemented in primary care, where it is likely that beliefs and expectations regarding treatment strategy are initially formed and where use of the tool can contribute to providing consistent education throughout the treatment process for OA patients.

There are several limitations of this study that need to be addressed. First, we included fewer patients than intended which impacted statistical power. The number of patients visiting our clinic with (suspicion) of OA was lower than expected which resulted in a slower inclusion rate. Due to time constraints inclusion was closed after 293 out of 382 intended patients were enrolled. Although loss to follow-up rates were lower than expected (7 and 25%, respectively), the final number of participants of whom data could be analyzed was slightly lower (*n* = 267 instead of *n* = 286) than aimed for based on sample size calculation. Considering the small differences found, it is not likely that we failed to detect an effect that was present (Type II error). Second, because the entire study was web-based we created bias against eHealth illiterate participants and may have excluded a relevant group of patients [40]. We did not systematically examine reasons for not participating because of practical reasons. Although gender and age of responders did not differ significantly from non-responders, further research into factors associated with participating in eHealth studies and use of the educational eHealth tool could provide starting points for improvement of the application [10, 39]. Last, we did not ask participants to discuss the tool with the specialist, but we cannot rule out that participants brought up the intervention during their consultations. Therefore, the orthopedic surgeons were not blinded in this trial, which might have changed the specialists’ behavior, outshining the effect of the educational eHealth tool.

## Conclusions

In this randomized controlled trial we demonstrated that an educational eHealth tool did not result in higher satisfaction with a first consultation in orthopedics outpatient clinic setting for patients with possible hip or knee OA. The eHealth tool did have small effects on knowledge and treatment beliefs. Future research is needed to evaluate if improving the educational eHealth tool and optimizing implementation in different care settings result in better outcomes.

## Data Availability

The dataset analyzed during the current study is available on the DANS repository (10.17026/dans-xjy-cuyw). In order to access the research data, researchers can register and login to the repository and put in a request to the authors. This article was written as part of a doctoral dissertation which can be found at https://repository.ubn.ru.nl/handle/2066/206304 [41].

## Abbreviations

OA: Osteoarthritis
TJR: Total joint replacement
BMI: Body mass index
WOMAC: Western Ontario and McMaster Universities Osteoarthritis Index
CQI: Consumer Quality Index
TOA: Treatment beliefs in OsteoArthritis questionnaire
CI: Confidence interval
IQR: Inter quartile range
SD: Standard deviation
PA: Physical activity
PM: Pain medication
